# On the natural history of Charcot-Marie-Tooth disease in children and adolescents

**DOI:** 10.1055/s-0045-1810624

**Published:** 2025-08-25

**Authors:** Francisco de Assis Aquino Gondim

**Affiliations:** 1 Universidade Federal do Ceará, Departamento de Medicina Clínica, Divisão de Neurologia, Fortaleza CE, Brazil.

Charcot-Marie-Tooth (CMT) disease was independently described in 1886 by Charcot and Marie in France and by Tooth in England. 1 2 Few years later, Déjerine and Sottas described severe infancy onset cases. 3 Soon, it also became clear that the disease was rather heterogenous, with major variation in inheritance patterns, onset, clinical course, prognosis and could also include very distinct features, such as tremor. 4

With the development of electromyography, CMT disease was subdivided according to nerve conduction velocities (CV)s into 1. CMT type 1: slow CVs or demyelinating form (median motor CV below 38 m/s); and 2. CMT type 2: median motor CVs above 38 m/s with decreased motor and sensory amplitudes or axonal form. 5 In the 90s, the most common form of CMT, i.e. CMT1A, was linked to a duplication within the chromosome 17p11.2. 6 In this new era of gene identification, the term CMT4 (or ARCMT1) has been employed to describe demyelinating autosomal recessive phenotypes and ARCMT2 for autosomal recessive axonal variants. To date, more than 110 genes have been linked to different CMT subtypes. Genetic studies have also evidenced that the past subdivision of demyelinating and axonal forms is not adequate to describe families with CVs in the intermediate range (later named DI-CMT). 7

In Brazil, studies about the epidemiology and evolution of the different subtypes of CMT are scarce, and important lacunes remain to be addressed by multicenter collaborative studies. Alvarenga Soares and colleagues 8 reported in this issue of Arquivos de Neuropsiquiatria the longitudinal findings of 3 consecutive evaluations of 30 children/adolescents with CMT (80% with CMT1A) seen at the Faculdade de Medicina de Ribeirão Preto over a 2-year period.

In this study, the participants were evaluated by the CMT Pediatric Scale (CMTPedS) and additional body indexes. In the CMT1A subgroup, the authors observed a 4-point increase in the CMTPedS score over 2 years (2 point increase per year). The functional dexterity test also showed a significant difference between evaluation one and evaluation three (p = 0.049) as well as the vibration sensation (p = 0.043) and gait scores (p = 0.048). Those results contrast with the 2.4 ± 4.9 point progression in the CMTPedS over 2-years observed in a large CMT Consortium study that included 206 participants. 9 It also differs from the 1.8 ±  4.2 point progression among 111 participants with CMT1A from the same study. 9 An increase in body mass index (BMI) was also observed in the results from Alvarenga Soares and colleagues, 8 which together with a possible type II error due to sample size differences, may at least partially explain the diverse findings. Overall, the study from Alvarenga Soares and colleagues 8 further emphasize the urgent need for additional multicenter genetic studies in Brazil and all over the world, including patients from different age groups and larger number of patients. Only with larger collaborative studies, we will be able to understand the clinical evolution/natural history and genotype-phenotype correlation of the different (and especially the more rare) CMT mutations. This subject is especially relevant, when one considers the ongoing prospects for the development of new different genetic treatments for CMT patients in the near future.
